# Supplementary material for: Spatial Distribution and Physicochemical Properties of Respirable Volcanic Ash From the 16–17 August 2006 Tungurahua Eruption (Ecuador), and Alveolar Epithelium Response In‐Vitro
Source: Geohealth. 2022 Dec 1;6(12):e2022GH000680. doi: 10.1029/2022GH000680 (PMC9758688; doi:10.1029/2022GH000680)
Supplement: Supplementary file 1 — Supporting Information S1 [file GH2-6-e2022GH000680-s001.pdf]

Spatial distribution and physicochemical properties of respirable volcanic ash from the 16-17 August 2006 Tungurahua eruption (Ecuador), and alveolar epithelium response *in-vitro*

Supplemental Material

Julia Eychenne[1,2*], Lucia Gurioli[1], David Damby[3], Corinne Belville[2], Federica Schiavi[1], Geoffroy Marceau[2,4], Claire Szczepaniak[5], Christelle Blavignac[5], Mickael Laumonier[1], Emmanuel Gardés[1], Jean-Luc Le Pennec[6,7], Jean-Marie Nedelec[8], Loïc Blanchon[2], Vincent Sapin[2,4]

[1] Université Clermont Auvergne, CNRS, IRD, OPGC, Laboratoire Magmas et Volcans, F-63000 Clermont-Ferrand, France

[2] Université Clermont Auvergne, CNRS, INSERM, Institut de Génétique Reproduction et Développement, F-63000 Clermont-Ferrand, France

[3] U.S. Geological Survey, California Volcano Observatory, Moffett Field, CA, USA

[4] Biochemistry and Molecular Genetic Department, University Hospital, F-63000 Clermont-Ferrand, France

[5] Université Clermont Auvergne, UCA PARTNER, Centre Imagerie Cellulaire Santé, F-63000 Clermont-Ferrand, France

[6] Geo-Ocean, CNRS, Ifremer, UMR6538, F-29280 Plouzané, France

[7] IRD Office for Indonesia & Timor Leste, Jalan Kemang Raya n°4, Jakarta 12730, Indonesia

[8] Université Clermont Auvergne, Clermont Auvergne INP, CNRS, ICCFn, F-63000 Clermont-Ferrand, France

Content of this file:

<u>Supplemental Figures S1 to S2 with captions</u>

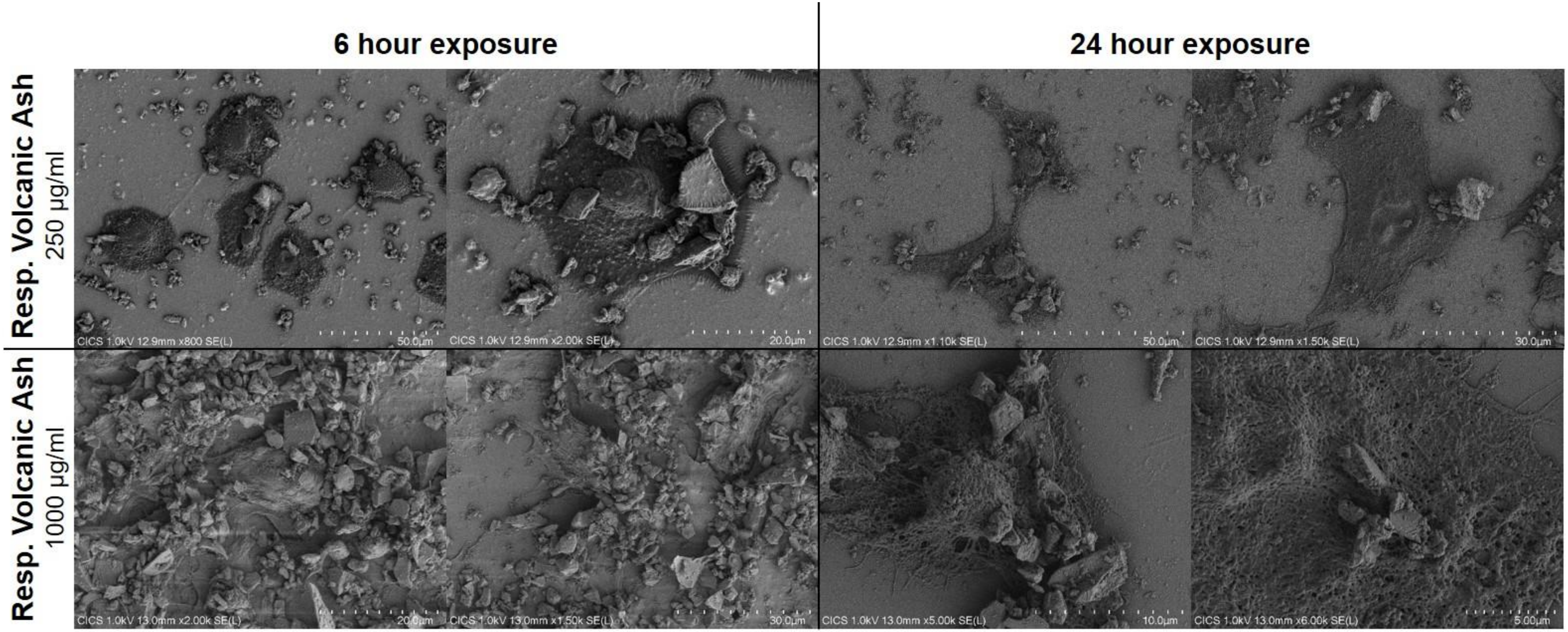

Fig. S1: Interactions between A549 cells and respirable Tungurahua volcanic ash at doses of 250 μg/ml and 1000 μg/ml after 6 and 24h of exposure, imaged by FEG-SEM.

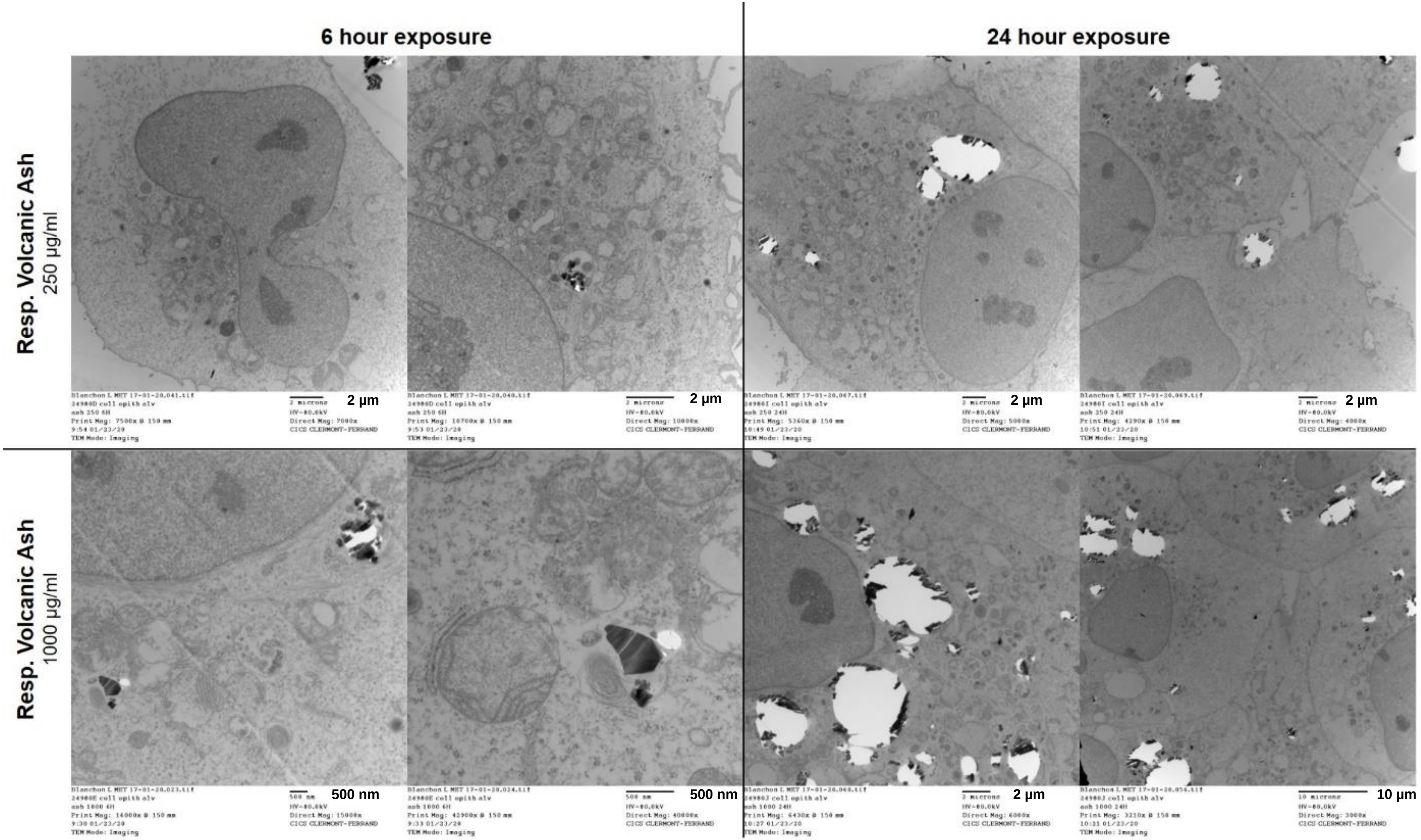

Fig. S2: Interactions between A549 cells and respirable Tungurahua volcanic ash at doses of 250 µg/ml and 1000 µg/ml after 6 and 24h of exposure, imaged by TEM.
